# IL‐15 Superagonist SHR‐1501 Enhances Immune Responses in Lung Cancer by Modulating Tumor Microenvironment

**DOI:** 10.1111/crj.70117

**Published:** 2025-08-13

**Authors:** Qian Zhang, Congli Hu, Minlin Jiang, Yuanyuan Wang, Heng Luo, Xuefei Li

**Affiliations:** ^1^ Department of Lung Cancer and Immunology, Shanghai Pulmonary Hospital School of Medicine, Tongji University Shanghai China; ^2^ Department of Pharmacy, Shanghai Pulmonary Hospital School of Medicine, Tongji University Shanghai China

**Keywords:** IL‐15 superagonist, mouse model, nonsmall‐cell lung cancer, PD‐1/PD‐L1 inhibitors, SHR‐1501

## Abstract

**Background:**

Interleukin‐15 (IL‐15) is a pleiotropic cytokine recognized as a promising therapeutic agent in cancer immunotherapy. IL‐15 superagonists have shown efficacy across various cancers, yet their effects in lung cancer immunotherapy remain underexplored.

**Methods:**

This study evaluated the antitumor effects of SHR‐1501 through intratumoral injection in two murine lung cancer models: Lewis lung carcinoma (LLC) and Kras G12D/p53−/− (KP). We employed flow cytometry to assess immune cell populations in the tumor microenvironment (TME) and systemic circulation. Immunohistochemistry (IHC) was used to analyze TME changes in tumor tissues, while single‐cell RNA sequencing provided insights into TME modulation following SHR‐1501 treatment. Additionally, we assessed the synergistic potential of combining SHR‐1501 with PD‐1 monoclonal antibody (mAb) therapy and explored the abscopal effect of SHR‐1501.

**Results:**

SHR‐1501 significantly inhibited tumor growth in both KP and LLC models at 5 μg and 15 μg doses (*p* = 0.0022 and *p* = 0.0002, respectively, for KP; *p* = 0.0508 and *p* = 0.0131, respectively, for LLC). Flow cytometry revealed increased infiltration of CD8+ T cells, effector memory CD8+ T cells (TEM), and natural killer (NK) cells in the TME. SHR‐1501 also enhanced systemic immune responses, increasing CD8+ T cells and TEM populations in peripheral blood and spleen, with an early NK cell elevation on day 7 post‐treatment. Single‐cell analysis indicated that SHR‐1501 promoted the activity of macrophages, increasing M1 macrophage proportions. Moreover, SHR‐1501 enhanced the antitumor immune response by promoting pro‐inflammatory changes across multiple cell types within the TME, including neutrophils, fibroblasts, and endothelial cells. When combined with PD‐1mAb, SHR‐1501 exhibited potent synergistic antitumor effects. The combination therapy significantly prolonged overall survival with no significant toxicity observed. Furthermore, SHR‐1501 may have the ability to induce an abscopal effect.

**Conclusion:**

SHR‐1501 demonstrated potent antitumor activity, especially when combined with PD‐1 mAb. Its mechanism likely involves promoting CD8+ T cell and NK cell infiltration and enhancing M1 macrophage activity. These findings provide evidence for further clinical trials exploring SHR‐1501 in nonsmall cell lung cancer (NSCLC) therapy.

## Graphical abstract

SHR‐1501 played an important role in regulating multiple important immune cell types and nonimmune cells with TME, which could shape the tumor immune landscape towards a pro‐immunogenic state. When combined with PD‐1mAb, SHR‐1501 exhibited superior antitumor efficacy.

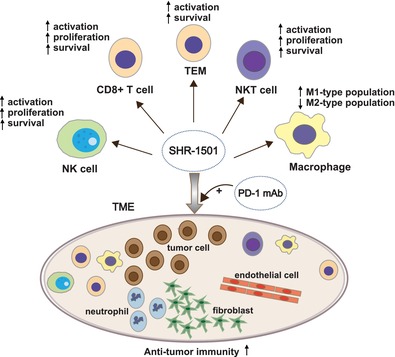

## 1 Introduction

Lung cancer remains a major global health concern, accounting for 85% of cases as nonsmall cell lung cancer (NSCLC) [1, 2]. In the past decades, there have been great advancements in immune checkpoint inhibitors (ICIs), specifically targeting the programmed death 1 (PD‐1) receptor and its ligand (PD‐L1) antibodies [3, 4, 5]. However, the low response rate and inevitable relapse have emerged as major concerns, limiting the long‐lasting clinical benefits of these ICIs [6]. Given the complexities of the tumor microenvironment (TME), researchers have explored immune‐modulatory cytokines as potential therapies [7]. Among them, interleukin‐15 (IL‐15) has emerged as a promising candidate due to its ability to enhance the cytotoxicity of immune cells, including CD8+ T cells and NK cells [8, 9, 10].

IL‐15 is a 14–15 kDa 4‐α‐helix bundle cytokine primarily secreted by dendritic cells, macrophages, and monocytes [11]. Its receptors are expressed on various types of immune cells, including natural killer (NK) cells, T cells, NKT cells, B cells, dendritic cells (DCs), and macrophages [12]. The IL‐15 receptor is a heterotrimeric structure comprising a specific IL‐15 receptor alpha subunit (IL‐15Rα), a beta subunit (IL‐15Rβ), and a gamma subunit (IL‐15Rγ) [13]. Among them, IL‐15Rα exhibits the highest affinity for IL‐15 [14]. Following IL‐15 binding to IL‐15Rα, the IL‐15/IL‐15Rαcomplex binds to IL‐15Rβ and ‐γ heterodimer expressed on effector T, B, and NK cells, thus potentiating the antitumor immunity of these effector cells [15]. IL‐15 has a strong ability to enhance the cytotoxic activity of CD8+ T cells and NK cells and promotes the production of cytotoxic effectors such as perforin, granzymes A and B, and interferon‐gamma (IFN‐γ) [12, 16, 17]. Upon the solid theoretical foundation of IL‐15 cytokine in immune responses, exploiting its function as an anticancer drug has shown great therapeutic potential.

However, the soluble form of IL‐15 (IL‐15 sol) has a short half‐life in vivo and is associated with a high incidence of toxicities. Some IL‐15 super‐agonists (IL‐15 SA) have been developed with a more advantageous structure. These IL‐15 SAs consist of a heterodimeric complex of IL‐15 and IL‐15Rα, which exhibits a more stable biological property and is associated with fewer adverse events (AEs) [18]. Several IL‐15SAs have been used in various cancers and have demonstrated significant anticancer efficacy in preclinical or clinical trials [18]. Accumulating evidence suggests that optimal IL‐15 SA efficacy as a therapeutic tool is achieved by combining it with other immune checkpoint inhibitors (ICIs), particularly PD‐1/PD‐L1 blockade therapy. Regarding NSCLC treatment, the combination strategy of IL‐15 cytokine with PD‐(L)1 blockade has also displayed promise [19]. In an open‐label, phase 1b clinical trial (NCT02523469), the combination of ALT‐803 with PD‐1 monoclonal antibodies (mAb) was assessed for safety and efficacy in NSCLC patients. The results support the antitumor efficacy of IL‐15 as a promising new class of agents in the treatment of NSCLC [19].

In this study, we used a novel IL‐15SA, SHR‐1501. It is a pharmacological grade IL‐15/IL‐15Rα complex fused to an IgG1 Fc, which is designed to extend IL‐1's half‐life and enhance its biological activity in vivo [20]. We investigated SHR‐150's antitumor effects alone and in combination with PD‐1 mAb in tumor‐bearing C57BL/6 mice models of lung cancer cell lines and explored how it modulates the TME. This is the first trial of this new drug on tumor‐bearing mice models of lung cancer. These preclinical findings provide evidence for further exploring SHR‐1501 as a promising anticancer therapeutic agent.

## 2 Results

### 2.1 SHR‐1501 Inhibited Tumor Growth in Both Tumor‐Bearing Mice Models

**FIGURE 1:**
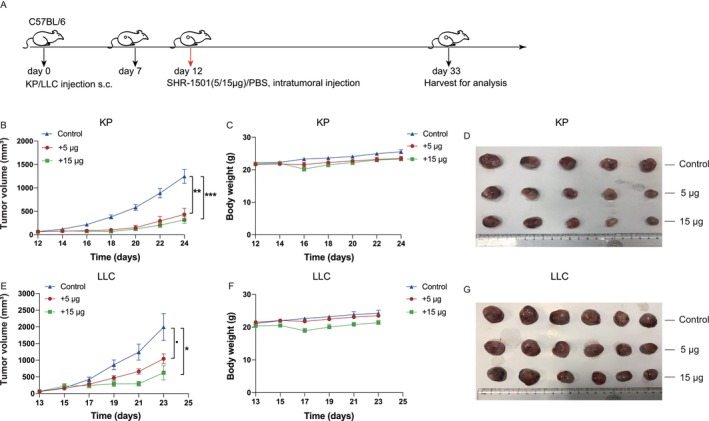
SHR‐1501 significantly inhibited tumor growth in both KP and LLC tumor‐bearing mice models. (A) Diagram of the treatment protocol in the subcutaneous tumor‐bearing mice models of LLC and KP lung cancer cell lines. Red arrow indicates administration of SHR‐1501. (B) Tumor growth curves of KP tumor‐bearing mice in each group following intratumoral administration of SHR‐1501 (*n* = 6 per group). (C) Body weight growth curves of KP tumor‐bearing mice in each group following intratumoral administration of SHR‐1501 (*n* = 6 per group). (D) Tumors were harvested from KP tumor‐bearing mice 21 days after SHR‐1501 administration and sorted within each treatment group from largest to smallest. (E) Tumor growth curves of LLC tumor‐bearing mice in each group following intratumoral administration of SHR‐1501 (*n* = 6 per group). (F) Body weight growth curves of LLC tumor‐bearing mice in each group following intratumoral administration of SHR‐1501 (*n* = 6 per group). Body weight and tumor volume were measured and recorded every other day throughout the experiment. (G) Tumors were harvested from LLC tumor‐bearing mice 21 days after SHR‐1501 administration and sorted within each treatment group from largest to smallest. Statistical significance was set at *p* < 0.05. “*” *p* < 0.05, “**” *p* < 0.01, “***” *p* < 0.001, “.” referred to *p* = 0.0508, and ns represents a *p*‐value indicating no statistical difference.

In this study, we investigated the therapeutic potential of SHR‐1501 in LLC and KP tumor‐bearing mice models. Treatment regimens were designed based on a previously published study of SHR‐1501 [21], administering comparable doses of 5 μg or 15 μg per mouse. Intratumoral injections of SHR‐1501 were initiated when the average tumor volume reached approximately 80 mm^3^. The treatment protocol is outlined in Figure 1A, where the red arrow denotes the administration of SHR‐1501.

Our continuous observation of tumor volume and body weight revealed that the novel drug SHR‐1501 significantly inhibits tumor growth in both KP and LLC tumor‐bearing mice models without causing significant toxicities. In the KP model, SHR‐1501–treated groups exhibited notably smaller tumor volumes compared to the control group (low‐dose: *p* = 0.0022; high‐dose: *p* = 0.0002) (Figure 1B). Additionally, there was no significant difference in body weight among the groups, indicating that the drug does not cause severe toxicities (Figure 1C). Tumors excised from each treatment group of KP mice were presented in Figure 1D.

In the LLC model, tumor volume was smaller in both SHR‐1501 treatment groups compared to the control group (low‐dose: *p* = 0.0508; high‐dose: *p* = 0.0131) (Figure 1E). There was no significant difference in body weight between the groups (Figure 1F), suggesting good tolerability of the treatment. Tumors harvested from each treatment group in the LLC tumor‐bearing mouse model are shown in Figure 1G.

In our study, we administered two different doses of the IL‐15 agonist SHR‐1501 to C57B/L6 mice bearing tumors: a low dose of 5 μg and a high dose of 15 μg per injection. We observed that in both models, the tumor volume in the high‐dose group was significantly smaller compared to the low‐dose group. This suggests that a higher dose of SHR‐1501 may elicit a more potent antitumor effect. Meanwhile, we observed that in the KP tumor‐bearing mice model, the tumors treated with low‐dose SHR‐1501 (5 μg per mouse) showed a highly significant reduction in tumor size, with an average decrease of 57%; however, in the LLC tumor‐bearing mice model, low‐dose SHR‐1501 treatment did not result in a similarly significant reduction in tumor size, with only a 24% reduction. This difference may indicate that the KP model is more sensitive to this new drug.

### 2.2 The Antitumor Effect of SHR‐1501 Is Achieved by Enhancing Effector CD8+ T Lymphocytes and NK Cells Infiltration in TME

To gain further insights into the mechanism underlying the antitumor effects of SHR‐1501, we examined the immune cell infiltration profiles within the tumor microenvironment (TME) using IHC staining and flow cytometry analysis.

**FIGURE 2:**
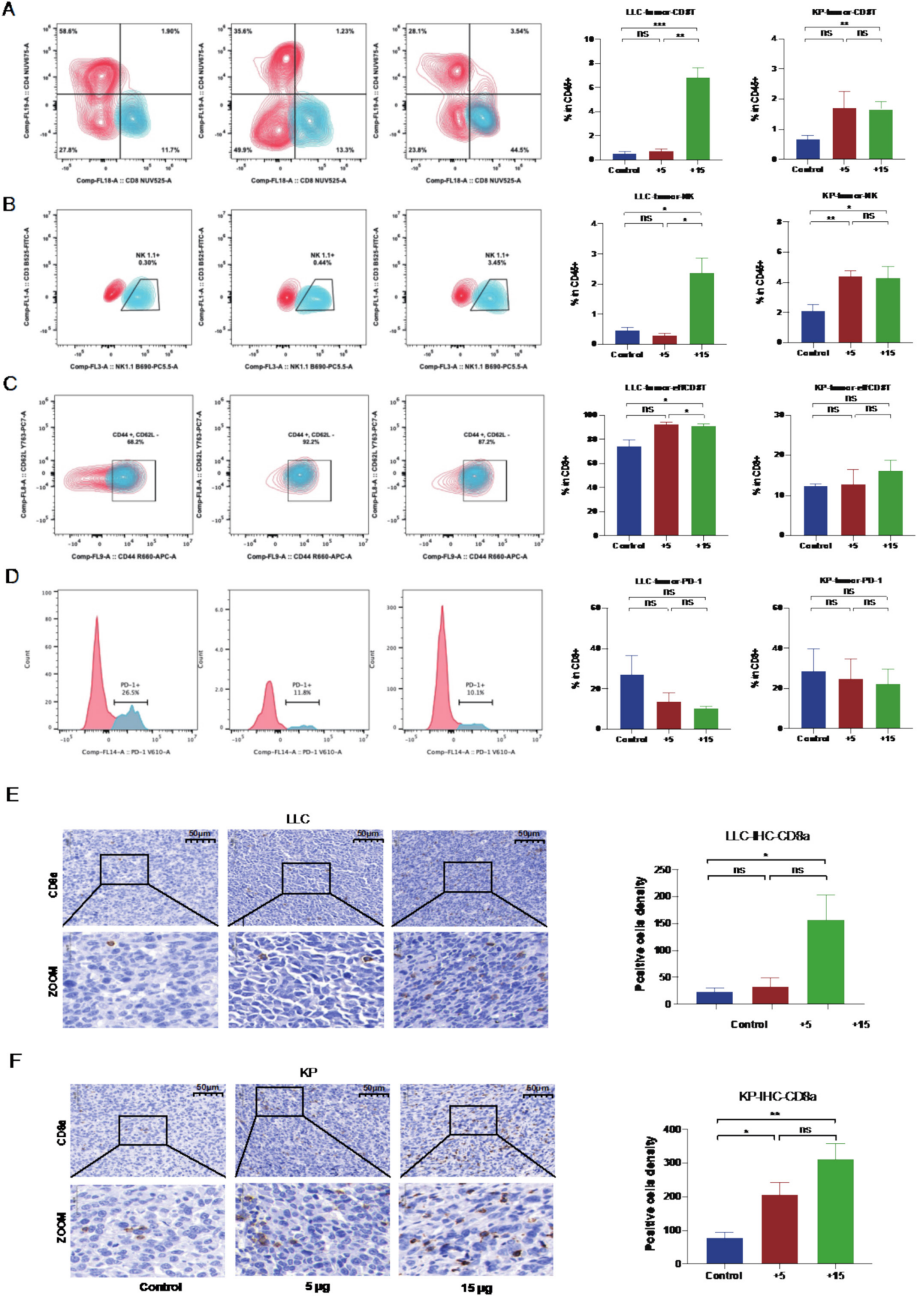
SHR‐1501 significantly increased the infiltration of CD8+ T cells and NK cells in the tumor microenvironment in both LLC and KP tumor‐bearing mice models. (A) Representative contour plots showing expression of CD8 T cells and bars depicting the proportions of CD8+ T cells in two tumor‐bearing mice models. (B) Representative contour plots showing expression of NK cells and bars depicting the proportions of NK cells in two tumor‐bearing mice models. (C) Representative contour plots showing expression of TEMs (CD44+, CD62L−) in CD8+ T cells subset and bars depicting the proportions of TEMs (CD44+, CD62L−) in CD8+ T cells subset in two tumor‐bearing mice models. (D) Representative histograms showing expression of PD‐1+ CD8+ T cells and bars depicting the proportions of PD‐1+ CD8+ T cells in CD8+ T cells subset in two tumor‐bearing mice models. (E) Representative IHC images depicting the expression of CD8a in tumor sections from LLC tumor‐bearing mice model. The bars depicting the density of CD8a positive cells observed in the IHC staining images. (F) Representative IHC images depicting the expression of CD8a in tumor sections from KP tumor‐bearing mice model. The bars depicting the density of CD8a positive cells observed in the IHC staining images. The positive cells density was measured as number/mm^2^. Statistical significance was set at *p* < 0.05. “*” *p* < 0.05, “**” *p* < 0.01, “***” *p* < 0.001, and ns represents a *p*‐value indicating no statistical difference.

First, we evaluated the proportions of immune cells CD4+ T cells, CD8+ T cells, effector CD8+ T cells, exhausted CD8+ T cells, and NK cells in TME by flow cytometry analysis. We found that the infiltrations of cytotoxic immune cell subsets, including CD8+ T cells and NK cells, were significantly enhanced in both high‐dose and low‐dose groups of SHR‐1501 treatment, as compared to the PBS control group (Figure 2A,B). Additionally, we observed a significant increase in the population of effector CD8+ T cells specifically (Figure 2C). The proportion of PD‐1+ CD8+ T cells showed a downward trend in the SHR‐1501 treatment group, although this did not reach statistical significance (Figure 2D). In both tumor models, IHC staining for CD8a indicates increased infiltration of CD8 T cells within the tumor microenvironment, as shown in Figure 2E,F. Our data demonstrate that SHR‐1501 treatment not only enhances the infiltration and recruitment of cytotoxic immune cell subsets but also promotes the activation of CD8+ T cells. These findings illustrate the antitumor effects of SHR‐1501 in two tumor‐bearing mice models of lung cancer cell lines.

As we have observed in the previous analysis of the tumor growth curve results, we found that in the KP model, the low‐dose treatment group exhibited a more pronounced increase in the number of CD8 T cells and NK cells. In contrast, in the LLC model, this increase was not as evident. These observations suggest that the KP model may be more responsive to this drug, as indicated by the greater increase in CD8 T cells and NK cells in the low‐dose treatment group compared to the LLC model.

### 2.3 Change of the Peripheral Immune System Confirms the Antitumor Effects of SHR‐1501

**FIGURE 3:**
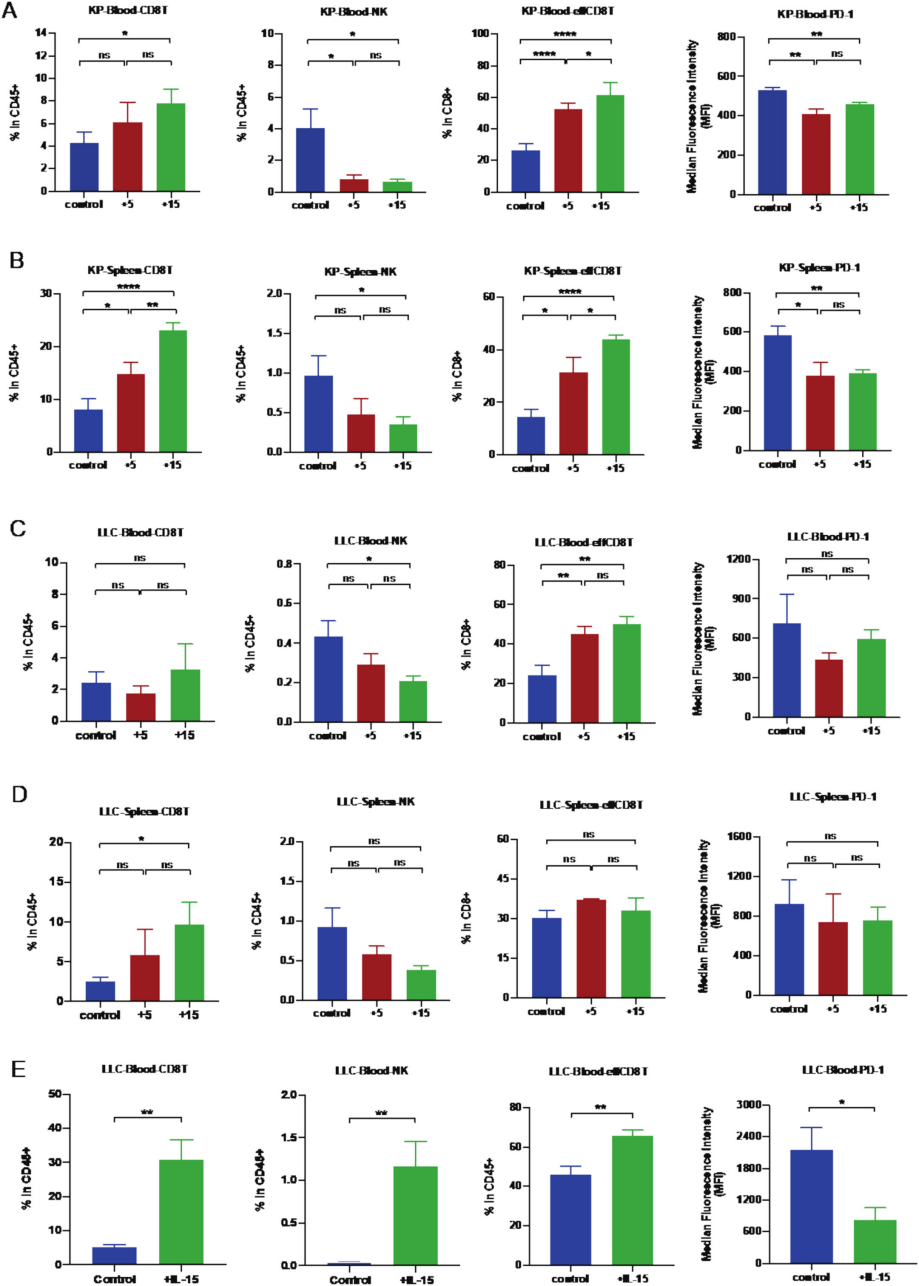
SHR‐1501 affected the ratio of CD8+ T and NK cells in peripheral immunity. (A) CD8+ T cell and NK cell frequency in blood samples harvested from KP tumor‐beraing mice model analyzed by flow cytometry. (B) CD8+ T cell and NK cell frequency in spleen samples harvested from KP tumor‐bearing mice model analyzed by flow cytometry. (C) CD8+ T cell and NK cell frequency in blood samples harvested from LLC tumor‐bearing mice model analyzed by flow cytometry. (D) CD8+ T cell and NK cell frequency in spleen samples harvested from LLC tumor‐bearing mice model analyzed by flow cytometry. (E) The frequency of CD8+ T cells and NK cells in blood samples harvested from LLC tumor‐bearing mice models at an early stage (7 days after SHR‐1501 administration) using flow cytometry analysis. All of the data in this study are derived from one experiment representative of two independent experiments. Statistical significance was set at *p* < 0.05. “*” *p* < 0.05, “**” *p* < 0.01, and “***” *p* < 0.001, and ns represents a *p*‐value indicating no statistical difference.

To further elucidate the antitumor mechanisms of SHR‐1501, we investigated alterations in the peripheral immune system. Blood and spleen samples were collected from both tumor‐bearing mice models at the end of the experiment to evaluate both systemic and localized immune responses. Flow cytometry analysis was performed to quantify changes in T cells and NK cells, including their subpopulations such as effector memory CD8+ T (TEM) and CD8+ PD1+ T cells. The results of these analyses are depicted in Figure 3.

In the KP tumor‐bearing model, SHR‐1501 treatment led to significant increases in both total CD8+ T cells and effector memory CD8+ T (TEM) cells in both the blood and spleen, as shown in Figures 3A,B. Notably, the high‐dose group exhibited more pronounced increases in these immune cell populations. Additionally, there was a significant decrease in exhausted CD8+ T cells (PD‐1+ CD8+) in peripheral blood, with the high‐dose group showing a more substantial reduction. In the LLC tumor‐bearing model, we observed similar changes in peripheral immune cell populations as those seen in KP models. Specifically, there were consistent increases in the frequencies of CD8+ T cells and effector memory T (TEM) cells, along with reductions in PD‐1+ CD8+ T cells (Figure 3C,D). These findings suggest that SHR‐1501 treatment enhances antitumor immunity by increasing the number of functional CD8+ T cells and reducing T cell exhaustion in both tumor‐bearing mice models.

However, we observed that SHR‐1501 treatment led to a decrease in NK cell levels in the peripheral immune system at the study's endpoint, with higher doses causing a more significant reduction. Simultaneously, there was an increase in the overall CD8+ T cell population, particularly in the TEM subset, and a reduction in exhausted T cells.

To explore early immune responses, blood and spleen samples were collected 7 days post‐treatment. Early changes in CD8+ T cells, TEM, and PD‐1+ CD8+ T cells were consistent with those observed at the study's endpoint. Notably, NK cell infiltration significantly increased at this early stage, indicating a rapid immune reaction to SHR‐1501 treatment (Figure 3E). This initial increase in NK cells contrasts with their later decrease in the peripheral system, suggesting a dynamic regulation of NK cell activity by SHR‐1501.

### 2.4 SHR‐1501 Enhances Macrophage Activity and Shapes the Tumor Immune Landscape Towards a Pro‐Immunogenic State

We employed single‐cell RNA sequencing to investigate the effects of SHR‐1501 on the tumor microenvironment (TME), analyzing a total of 15 716 cells, including 6897 cells from the SHR‐1501–treated group and 8819 cells from the control group (Figure S1A).

**FIGURE 4:**
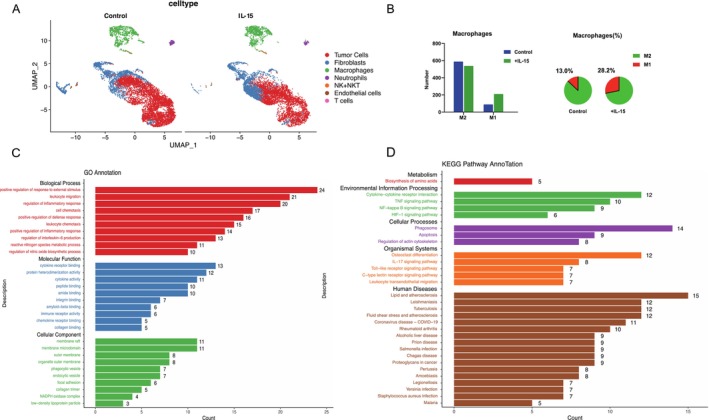
SHR‐1501 enhanced antitumor immunity of macrophages within TME. (A) UMAP of total collected cells from LLC tumor samples. (B) Quantity and proportion of M1, M2 macrophage subtypes by single‐cell analysis. (C) GO enrichment analysis of macrophages. (D) KEGG enrichment analysis of macrophages.

Across both groups, excluding tumor cells, fibroblasts were the most abundant population, followed by macrophages (Figure 4A). In the control group, the cellular composition was: tumor cells (62%), fibroblasts (24.1%), macrophages (11.2%), neutrophils (1.7%), endothelial cells (0.7%), and NK(T)+ T cells (0.2%). In contrast, the SHR‐1501‐treated group exhibited the following proportions: tumor cells (54.5%), fibroblasts (30.9%), macrophages (13.1%), neutrophils (0.8%), endothelial cells (0.2%), and NK(T)+ T cells (0.5%). Compared to the control group, the SHR‐1501 treatment group exhibited increased T cell and NK cell proportion, aligning with previous flow cytometry results (Figure S1B,C). Given the reported effects of IL‐15 agonists on macrophage activity, we further focused on macrophages, which were the largest number of immune cell types in our analysis. SHR‐1501 treatment significantly increased the proportion and absolute number of M1 macrophages (Figure 4B).

Gene Ontology (GO) analysis of macrophages from the SHR‐1501–treated group identified enrichment in pathways related to leukocyte migration, positive regulation of inflammatory responses, cytokine receptor binding, and cytokine activity. In addition, metabolism‐related pathways, including ATP metabolic processes and oxidative phosphorylation, were enriched (Figure 4C). Kyoto Encyclopedia of Genes and Genomes (KEGG) analysis further revealed positive enrichment in the TNF signaling pathway, NF‐κB signaling pathway, and IL‐17 signaling pathway (Figure 4D). These results suggest that SHR‐1501 shifts the TME toward a pro‐inflammatory and immune‐supportive state, enhancing macrophage‐mediated antitumor activity. We also performed GO and KEGG pathway analyses on fibroblasts, neutrophils, and endothelial cells. Notably, endothelial cells exhibited enriched pathways associated with type I interferon production and interleukin‐1 beta production (Figure S2). Fibroblasts in the SHR‐1501–treated group showed significant enrichment in MHC class I‐related pathways, including the MHC class I protein complex and peptide loading complex (Figure S3). In neutrophils, pathways related to mRNA processing were significantly enriched following SHR‐1501 treatment (Figure S4).

These findings suggest that SHR‐1501 enhances the antitumor immune response by promoting pro‐inflammatory changes across multiple cell types within the TME. The observed increase in M1 macrophages, combined with the activation of critical immune pathways, supports the potential of SHR‐1501 to reprogram the immune landscape toward more favorable conditions for antitumor immunity.

### 2.5 SHR‐1501 in Combination With PD‐1 Monoclonal Antibody Exhibited Superior Antitumor Efficacy

**FIGURE 5:**
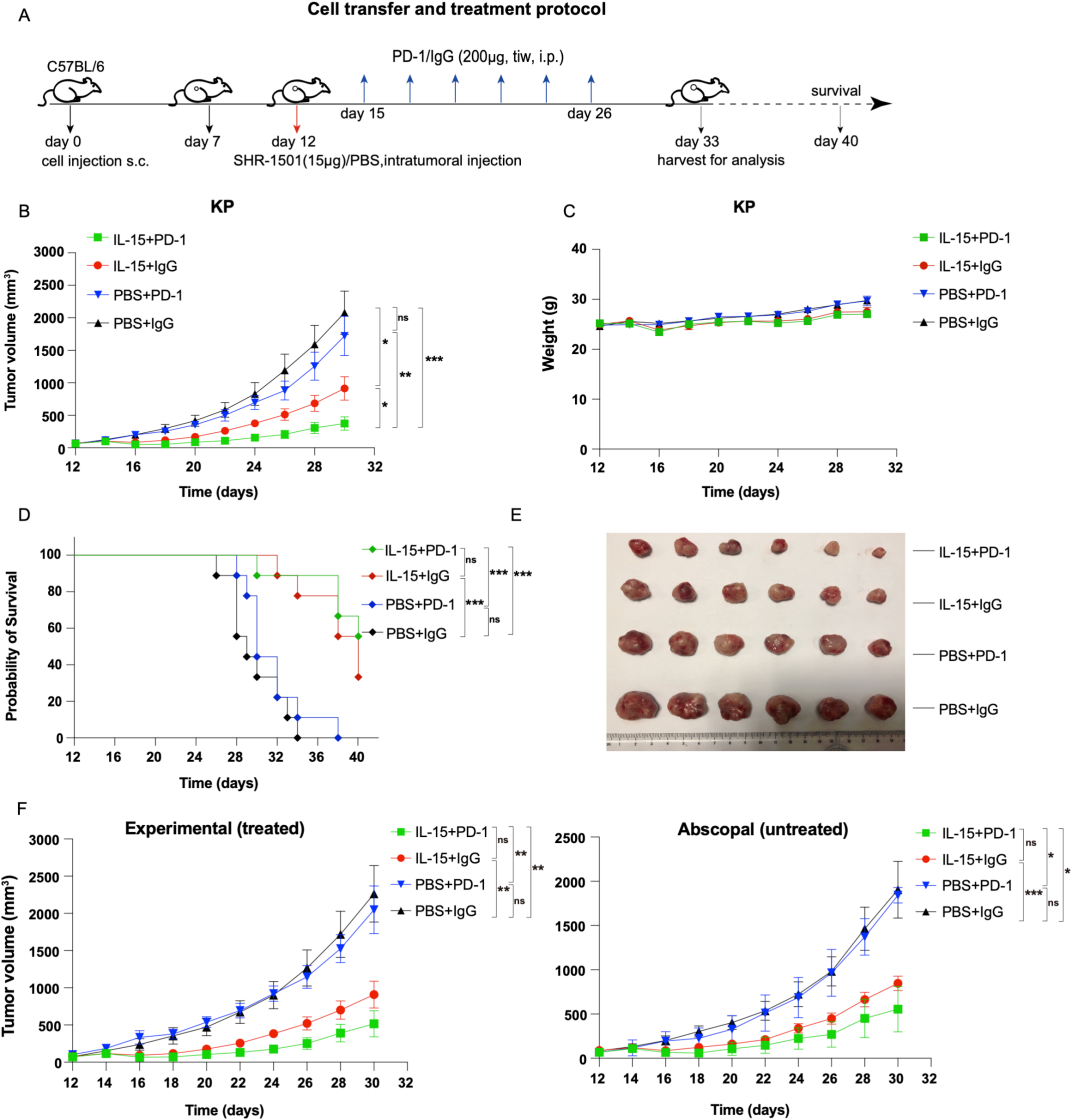
The combination of SHR‐1501 with PD‐1 mAb had shown a potent synergistic antitumor effect. (A) Cell transfer and treatment protocol in the subcutaneous tumor‐bearing mice model of KP lung cancer cell line. Red arrow indicates administration of SHR‐1501. Blue arrows indicate intraperitoneally injection of PD‐1 mAb. (B) The tumor growth curves in each treatment group following intratumoral injection of SHR‐1501 (*n* = 6 per group). The tumor volume was measured every other day. (C) Body weight growth curves in each group following intratumoral injection of SHR‐1501 (*n* = 6 per group). The body weight was measured every other day. (D) Survival curves of each group from an independent experiment starting from the day of SHR‐1501 administration (*n* = 9 per group). (E) The tumors harvested from each treatment group 21 days after the administration of SHR‐1501 and were sorted from largest to smallest within their respective treatment groups. (F) In the tumor models constructed for abscopal effect experiment, mice were bilaterally tumor‐bearing. The experimental tumors underwent intratumoral injection of SHR‐1501, wheras the abscopal tumors did not. The growth curves of experimental tumors and abscopal tumors were simultaneously observed (*n* = 3–5 per group). Statistical significance was set at *p* < 0.05. “*” *p* < 0.05, “**” *p* < 0.01, and “***” *p* < 0.001, and ns represents a *p*‐value indicating no statistical difference.

Following the encouraging antitumor activity observed with SHR‐1501, we proceeded to evaluate its synergistic potential in combination with a PD‐1 monoclonal antibody (mAb) in a KP tumor‐bearing mouse model. In this combination therapy study, SHR‐1501 was administered at a dose of 15 μg per mouse via intratumoral injection, while the PD‐1 monoclonal antibody was given at 200 μg per mouse through intraperitoneal injection. The treatment regimen involved a single administration of SHR‐1501 combined with six administrations of the PD‐1 monoclonal antibody, as outlined in Figure 5A.

The combination therapy group (SHR‐1501 plus PD‐1 mAb) demonstrated significant inhibition of tumor growth (Figure 5B), exhibiting the slowest tumor growth rate among all treatment groups. Weight loss in the SHR‐1501–treated groups did not exceed 10% of the control group's body weight, and there was no statistically significant difference compared to the control group (Figure 5C). This suggests that the combination treatment was well tolerated. Additionally, the combination therapy significantly prolonged overall survival compared to single‐agent treatments (Figure 5D). Tumors harvested from each treatment group in the KP tumor‐bearing mouse model were shown in Figure 5E. These results indicated that the combination of SHR‐1501 with PD‐1 mAb provides superior antitumor efficacy over monotherapy with either agent.

To further explore the therapeutic potential of SHR‐1501, we employed a bilateral tumor‐bearing mouse model to assess whether SHR‐1501 could induce an abscopal effect. The abscopal effect refers to a systemic immune response initiated by localized treatment, resulting in the suppression of distant, untreated tumors [22]. In our study, SHR‐1501 was administered via intratumoral injection to one tumor site, and we observed tumor repression at the contralateral site, which had not received direct SHR‐1501 treatment (Figure 5F). Our findings suggest that SHR‐1501 elicits a systemic immune response capable of targeting distant tumors, which is indicative of an abscopal effect. We hypothesized that this effect may be related to SHR‐1501–induced changes in the peripheral immune system. These changes, in turn, may lead to similar tumor shrinkage effects in remote, unadministered tumors through peripheral immune mediation. However, further experiments are required to validate and confirm our results.

## 3 Discussion and Limitation

IL‐15 is a promising cytokine for treating cancer and viral diseases. An NCI review listed IL‐15 as the most promising candidate among 12 immunotherapy drugs that could potentially cure cancer [23]. To date, the administration of recombinant human IL‐15(rhIL‐15) and IL‐15 agonists has been validated by multiple preclinical and clinical trials in various types of cancers [22] but is limited in lung cancer immunotherapy. In our study, we demonstrated that SHR‐1501 is a potent and effective IL‐15SA with improved biological activity and therapeutic potential in lung cancer treatment. This is the first experimental study of the novel drug administered to lung cancer tumor‐bearing mice models.

In our study, we employed flow cytometry, immunohistochemistry (IHC), and single‐cell analysis to analyze tumor samples. The results revealed that SHR‐1501 administration led to increased infiltration of CD8+ T cells, particularly effector memory CD8+ T cells (TEM), and NK cells within the TME. Additionally, SHR‐1501 enhanced the proportion of total CD8+ T cells, TEM cells, and NK cells in peripheral blood and spleen. These findings suggested that SHR‐1501 exerts antitumor effects by modulating both the tumor microenvironment and systemic immunity. Consistent with our findings, a clinical study on cytokine copy number variations reported that IL‐15 deficiency was correlated with shorter disease‐free survival (DFS) and reduced intratumoral infiltration of total, activated, and cytotoxic T cells, as well as Th1, memory T, and NK cells in colorectal cancer [24]. In a phase I clinical trial of patients with metastatic malignancy, the administration of rhIL‐15 could significantly alter the homeostasis of lymphocyte subsets in the blood, with a marked expansion of NK cells and memory CD8 T cells [25]. Similarly, in other clinical trials for advanced solid tumors, IL‐15 administration was associated with increased circulating NK and effector CD8 T cells [26]. Our results align with these prior researches, underscoring the potent antitumor efficacy of SHR‐1501 as a novel IL‐15 super‐agonist in tumor‐bearing mice models of lung cancer cell lines.

The flow cytometry analysis of TME showed that the expression of PD1 is limited to a small subset of CD8 cells, but the combination of the anti–PD‐1 and SHR‐1501 can lead to a quite evident therapeutic effect. A latest study in 2023 proposed that high PD‐1/PD‐L1 interaction can serve as a predictive biomarker for stratification in NSCLC patients. When there is a high interaction between PD‐1 and PD‐L1, even if PD‐L1 is lowly expressed (TPS < 24%), it may still be effective for PD‐1/PD‐L1 immunotherapy [27]. Furthermore, another published study has demonstrated that IL‐15 treatment can significantly enhance the effect of anti–PD‐1 antibodies by enhancing the proliferation of CD28− PD‐1+ CD8+ TILs as well as CD28+ PD‐1+ CD8+ TILs. These results provide mechanistic insights into combining IL‐15 with anti–PD‐1 antibodies [28].

As we know, IL‐15 monomer has been limited due to its short half‐life and suboptimal efficacy. Upon binding to its high‐affinity receptor α, the IL‐15/IL‐15Rα complex exhibits an extended half‐life and enhanced efficacy in vivo due to increased stability. Multiple IL‐15 agonists, such as RLI, ILR, NIZ985, and pro‐IL‐15, have already been utilized in the treatment of different tumor models [29, 30]. For example, NIZ985 in a preclinical study could enhance pancreatic tumor sensitivity to αPD‐1, with improved antitumor and survival benefits [31]. RLI restored the balance between NK cells and neutrophils (CD11b+ Ly6Ghigh Ly6Clow) that massively infiltrate the lungs of 4T1–tumor bearing mice. In addition, the ratio between NK cells and Treg was strongly increased by RLI treatment [32]. As we described above, these IL‐15SAs consist of a heterodimeric complex of IL‐15 and IL‐15Rα. In our experimental study, we employed a novel IL‐15 superagonist (IL‐15SA), SHR‐1501. SHR‐1501 has superior structural features compared to them. It is a pharmacological grade IL‐15/IL‐15Rα complex fused to an IgG1 Fc. By binding to the soluble dimer IL‐15 Rα/IgG fusion protein, its activity can be further enhanced to 10–25 times that of natural IL‐15 [33]. The advantage of this structure of SHR‐1501 is that it makes IL‐15 binding more stable compared to other IL‐15SA structures and allows IL‐15SA to exert a greater biological effect.

In addition to the IL‐15SAs mentioned above, another IL‐15SA called ALT‐803 exhibits a structure most similar to SHR‐1501 [33]. ALT‐803 and SHR‐1501 both consist of IL‐15 molecules and a dimeric IL‐15Rα sushi domain‐IgG1 Fc fusion protein. The difference lies in that ALT contains a mutant form of the IL‐15 molecule (IL‐15 N72D mutated), whereas SHR contains the natural form of the IL‐15 molecule [12]. However, no conclusive comparative trials have been performed on the biological effects between them. Besides, these two drugs also show great similarities in clinical application; both of them can be used in the field of lung cancer immunotherapy. ALT‐803 has been approved by the FDA for use in combination with BCG in the treatment of nonmuscle‐invasive bladder cancer [34]. SHR‐1501 is currently approved for clinical use in combination with ICIs in the treatment of bladder cancer, like ALT‐803 [35]. But their clinical administration routes differ; for example, in bladder cancer, ALT‐803 was administered by subcutaneous injection, whereas SHR‐1501 was administered by bladder perfusion. These different administration routes may lead to variations in drug distribution and exposure, thus making it difficult to directly compare their clinical efficacy.

In our study, SHR‐1501 was administered via intratumoral injection. However, for its potential application in clinical trials for NSCLC immunotherapy, it is crucial to optimize the route of administration. One promising approach could be subcutaneous injection, similar to the method used for ALT‐803 in NSCLC clinical trials [19]. Further preclinical and clinical studies are warranted to fully evaluate its safety and efficacy in human patients.

Our study has several limitations. First, single‐cell RNA sequencing suggests an increase in M1‐type macrophages in the TME with SHR‐1501; we still need to further confirm the results of single‐cell sequencing by flow detection and IHC in future experiments. Then, our study has confirmed the antitumor efficacy of this new drug in vivo using animal models. To enhance the credibility of our findings and strengthen the overall validity of the experiment, it is essential to conduct additional validation tests in vitro to further assess the effects of this drug.

Third, to gain deeper insights into the functional capabilities of CD8 T cells and NK cells in our flow cytometry studies, functional markers such as Granzyme B, Perforin, and IFN‐γ will be included in our flow cytometry analyses of CD8 T cells and NK cells to assess their cytotoxic potential and inflammatory activity.

In conclusion, SHR‐1501 shows significant promise as a novel immunotherapy for NSCLC, particularly when combined with PD‐1 inhibitors. More in‐depth experiments are required to elucidate the underlying mechanisms and assess the safety profile of this combination treatment. These findings lay the groundwork for further exploration of the therapeutic potential of SHR‐1501 in lung cancer treatment.

## 4 Materials and Methods

### 4.1 Cell Lines and Reagents

Lewis lung cancer (LLC) and Kras G12D, p53−/− (KP) lung cancer cell lines were kindly provided by Prof. Fei Li at Fudan University. Cell lines were maintained in DMEM (Gibco) supplemented with 10% fetal bovine serum (FBS) (Gibco) and 1% penicillin–streptomycin (Gibco). SHR‐1501 for injection (1 mg/vial) and anti–PD‐1 mAb (HRP00262‐022) were generously provided by Jiangsu Hengrui Pharmaceuticals Co. Ltd. under a cooperative research and development agreement with the National Cancer Institute. RecombiMAb human IgG4 (S228P) isotype control (clone: N/A‐CP147, BioXcell, CP147‐50MG) was purchased from BioXcell (West Lebanon, New Hampshire, USA). The appearance of SHR‐1501 for injection (1 mg/vial) is a white to almost white block or powder, dissolved with 1.1 mL sterile PBS liquid, and divided into corresponding small droplets and stored at −80°C for use.

### 4.2 Animal Model and Experiments

Male C57BL/6 mice, aged 5–6 weeks, were purchased from Shanghai SLAC Laboratory Animal Co. Ltd. (Shanghai, China), and maintained at the Experimental Animal Center (Shanghai Pulmonary Hospital, China) under specific pathogen‐free conditions in accordance with the Association for Assessment and Accreditation of Laboratory Animal Care (AAALAC) guidelines. Prior to use in experiments, the mice were acclimatized to the Experimental Animal Center for a week.

All cell lines were cultured according to Prof Li's instructions. The tumor volume and body weight were measured every other day using calipers and a mouse weight scale. Tumor volume (mm^3^) was calculated using this formula: (length × width^2^)/2. The body weight of the mice was measured using a specialized weight scale designed for mice. These measurements allowed for the monitoring of tumor growth and body weight change throughout the study. In the subcutaneous tumor model, a total of 1 × 10^6^ LLC or KP cells were resuspended in 100 μL phosphate buffered saline (PBS) and inoculated subcutaneously into the right flank of each male C57BL/6 mouse. Approximately 7 days after the inoculation, a palpable tumor can be felt subcutaneously. Mice were then randomly assigned to different treatment groups based on their tumor volume. When the tumors reached a specific volume range of 80 mm^3^, the administration of SHR‐1501 intratumoral injection was carried out. In the abscopal effect experimental tumor model, mice were bilaterally tumor‐bearing; therefore, the tumor cell suspensions were inoculated subcutaneously into both the right and left flanks of each mouse.

The mice were administered a single dose of SHR‐1501 via intratumoral injection at either 5 or 15 μg per mouse. To ensure consistency in the total amount of intratumoral injection, we adjusted different effective doses of IL‐15 to a total volume of 50 μL using appropriate solvents for each intratumoral injection. In SHR‐1501 combination therapy, administration of PD‐1 monoclonal antibody (PD‐1 mAb) at a dose of 200 μg/day per mouse, 3 days a week for 2 weeks. When the tumor volumes in the control group reached 2000 mm^3^, the tumor‐bearing mice were anesthetized, and all tissues, including tumor, blood, and spleen, were harvested for further analysis and measurement. Survival analysis was conducted as independent experiments for indicated days. All the animal studies were conducted in compliance with the institutional guidelines and were approved by the Institutional Committee for Animal Care and Use at Shanghai Pulmonary Hospital.

### 4.3 Flow Cytometry Analysis and Antibodies

Tumors, blood, and spleen samples were harvested from the experimental mice on the indicated days. Tumor samples were cut into small pieces and enzymatically digested using the single‐cell suspensions of tumor tissue generated with a tumor dissociation kit (mouse, Miltenyi Biotec) according to the manufacturer's instructions. Spleen samples were digested into single‐cell suspensions by mechanical dissociation. Blood samples were lysed in Red Blood Cell Lysis Buffer (TianGen) for 15 min and washed twice with DPBS. Firstly, reserve a tube of the cell suspension without any staining. The cell suspension was suspended in 1 mL DPBS and stained with 1 μL FVS 510 to distinguish between deceased and viable cells. Then, it was washed twice with stain buffer. To prevent nonspecific staining, single‐cell suspensions underwent Fc Blocking with CD16/CD32 (mouse BD Fc Block, clone: 2.4G2, BD Pharmingen: 553141) for 15 min prior to surface antigens staining. Cells were then stained with the following Abs for 30 min at 4°C: CD45 (APC‐CY7, clone: 30‐F11, BD Pharmingen: 557659), CD3 (FITC, clone: 145‐2C11, BD Pharmingen: 553061), CD4 (BUV661, clone: RM4‐5, BD Pharmingen: 741461), CD8 (BUV496, clone: 53‐6.7, BD Pharmingen:750024), CD62L (PE‐Cy7, clone: MEL‐14, BD Pharmingen: 560516), CD44 (APC, clone: IM7, BD Pharmingen: 559250), NK1.1 (BB700, clone: PK136, BD Pharmingen: 556502), CD69 (BV786, clone: H1.2F3, BD Pharmingen: 564683), CD223 (BV786, clone: C9B7W, BD Pharmingen: 740959), CD279 (PD‐1) (BV605, clone: J43, BD Pharmingen: 563059), and CD366 (TIM‐3) (BV421, clone: 5D12/TIM‐3, BD Pharmingen: 747626) The flow cytometry data were acquired on the Beckman CytoFLEX LX Flow Cytometer (Beckman Coulter, Model NO.: B90883) and analyzed with FlowJo 10.8.1 software.

### 4.4 Tissue Dissociation and Single‐Cell Suspension Preparation

Fresh tumor samples from experimental and control mice groups were collected in the tube with Tissue Preservation Solution (Miltenyi Biotec) at 4°C. The samples were minced into 1 mm^3^ fragments with sterile scalpels, and then suspended in 5 mL of digestion buffer consisting of the digestion buffer [RPMI 1640 medium (Coring, 10‐040‐CV), collagenase type II (2 mg/mL, Sigma, C6885) and DNase I (200 U/mL, Worthington, LS006344)], and incubated with shaking for 45 min at 37°C. Then, the suspension was passed through filters (100 μm, Falcon, 3 523 260) and centrifuged at 400 *g* for 10 min at 4°C. Pelleted cells were resuspended in red blood cell lysis buffer (Solarbio, R1010) repeating filtering (Falcon, 3 523 240) and centrifuging (400 *g*, 10 min, 4°C), and re‐suspended in PBS (BI, 02‐024‐1ACS) containing 0.04% BSA (Sigma, B2064). AO‐PI (LUNA, D23001) Trypan blue (Thermo, T10282) were used for cell counting. Using the Chromium Controller (10X Genomics) to process single cells based on the manufacturer's protocol.

### 4.5 Single‐Cell Sequencing and Data Processing

Single‐cell 3′ gene expression profiling was processed using Chromium Next GEM Chip G Single Cell Kit (10x Genomics, 1 000 120) and Chromium Next GEM Single Cell 3′ Kit v3.1 (10x Genomics, 1 000 268). Chromium single cell controller (10x Genomics) was used for the generation of single‐cell gel beads and Illumina NovaSeq6000 system was used to sequence the 3′ gene expression libraries of cell barcodes in Shanghai Biochip Co. Ltd. (Shanghai, China).

Reference genome of mouse (refdata‐gex‐mm10‐2020‐A) was used to process the raw data. We used Seurat (version 4.0.3) R package for the identification of distinct cell clusters. Genes that expressed ≥ 3 cells were selected. Cells with mitochondrial gene expression lower than 25% were included for further analysis. NormalizeData function was used for the normalization of raw data, and FindVariableFeatures function for the extraction of highly variable genes. Then, the top 3000 highly variable genes were extracted and principal component analysis (PCA) was performed. After cluster analysis by using the significant principal components (top 30), t‐distributed stochastic neighbor embedding (t‐SNE) and uniform manifold approximation and projection (UMAP) were used for visualization. Seurat FindMarkers function was used for the identification of marker genes. GO and KEGG enrichments were conducted for enrichment analysis based on clusterProfiler software. All visualization was implemented using R package.

### 4.6 Immunohistochemistry (IHC)

Immunohistochemistry assays were conducted to investigate the alterations in the TME in mice tumor tissues, with a specific focus on the infiltration of CD8+ T cells. Formalin‐fixed paraffin‐embedded (FFPE) specimens were cut into 3 μm sections. The sections were deparaffinized first with an environmentally friendly dewaxing solution (3 times, 10 min each) and then with anhydrous ethanol (3 times, 5 min each), and washed in distilled water. Sections were submerged into acid antigen repair buffer and microwaved for antigen repair. For blocking endogenous peroxidase activity, the sections were placed in a 3% hydrogen peroxide solution (at room temperature, 25 min), and washed with PBS on a decolorizing shaker (3 times, 5 min each). This was followed by covering with 3% BSA for serum closure (at room temperature, 30 min). Slides were incubated with CD8 (1:2000, Abcam, ab217344) antibody in a wet box at 4°C overnight. Prior to secondary antibody incubation, the slides were washed with PBS on the decolorizing shaker (3 times, 5 min each). The slides were covered with HRP‐labelled goat antirabbit secondary antibody (GB23303, 1:200) and incubated (at room temperature, 50 min). After washing on the decolorizing shaker with PBS (3 times, 5 min), DAB color developing solution was added to the slides for color developing, and the positive color was brown and yellow under the microscope. For re‐staining nuclei, the slides were re‐stained with hematoxylin (3 min) and hematoxylin differentiation solution for a few seconds, and the process was rinsed with tap water. The nucleus color was blue. Finally, alcohol (75%, 85%, 5 min each), anhydrous ethanol (3 times, 5 min) and xylene (once, 5 min) for dehydration and transparency. The IHC results are interpreted by a microscope. The number of image capture processes was performed by a Digital Slide Scanner (Pannoramic MIDI, Pannoramic 250FLASH, Pannoramic DESK) and analyzed by Aipathwell software. Positive cell density was adopted for immune cell counting and recorded as numbers/mm^2^.

## 5 Statistics

All statistical analyses were performed in Prism 9.0.0 (macOS). Unless otherwise specified, data presented in bar graphs or scatter plots were analyzed using one‐way ANOVA with Tukey's multiple comparisons test. Two‐way or ordinary ANOVA was used to analyze tumor growth curves. The survival experiment was analyzed using the log‐rank (Mantel‐Cox) test. Outliers were identified using the ROUT test. Statistical significance was set at *p* < 0.05. “*” referred to *p* < 0.05, “**” referred to *p* < 0.01, and “***” referred to *p* < 0.001, and ns represents a *p*‐value indicating no statistical difference.

## Author Contributions

Q.Z. designed this study and drafted the manuscript. C.H. and M.J. collected flow cytometry data. Q.Z. and Y.W. analyzed the data. H.L. and X.L. conception and design, funding support, critical revision of the article, and final approval of the article. All authors contributed to this article and approved the submitted version.

## Ethics Statement

The study was approved by the ethics committee of Shanghai Pulmonary Hospital (No. K21‐313Z).

## Conflicts of Interest

The authors declare no conflicts of interest.

## Supporting information


**Figure S1:** Single‐cell analysis provided additional validation to the findings from flow cytometry. (A) The image of physical tumors collected from experiments. (B) Single cell analysis of the two major classes of immune cell types. (C) The cellular characteristics of the TME by single‐cell analysis.

**Figure S2:** GO analysis for endothelial cells.

**Figure S3:** GO analysis for fibroblasts.

**Figure S4:** GO analysis for neutrophils.

**Data S1:** Supplementary Information.

## Data Availability

Research data are not shared.
