# Supplementary figures and images for: IL‐15 Superagonist SHR‐1501 Enhances Immune Responses in Lung Cancer by Modulating Tumor Microenvironment
Source: Clin Respir J. 2025 Aug 13;19(8):e70117. doi: 10.1111/crj.70117 (PMC12344376; doi:10.1111/crj.70117)

A

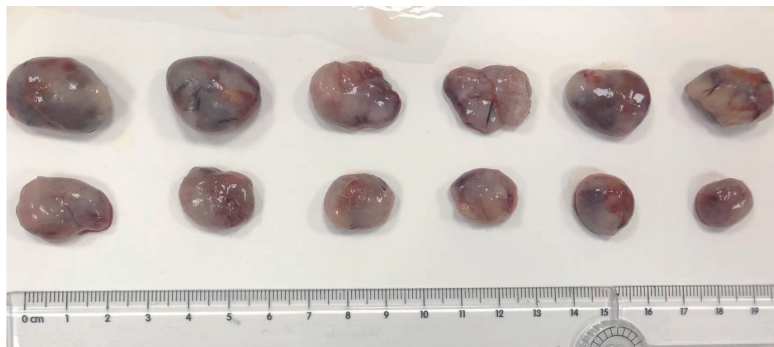

B

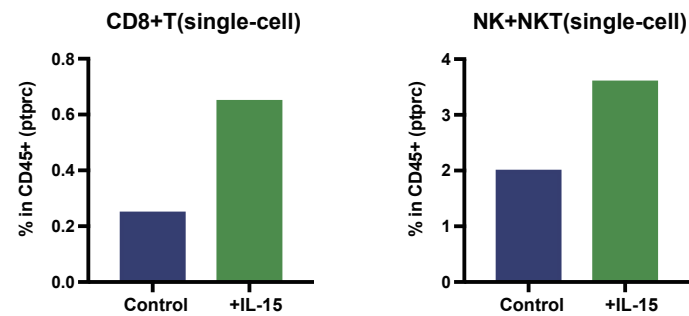

C

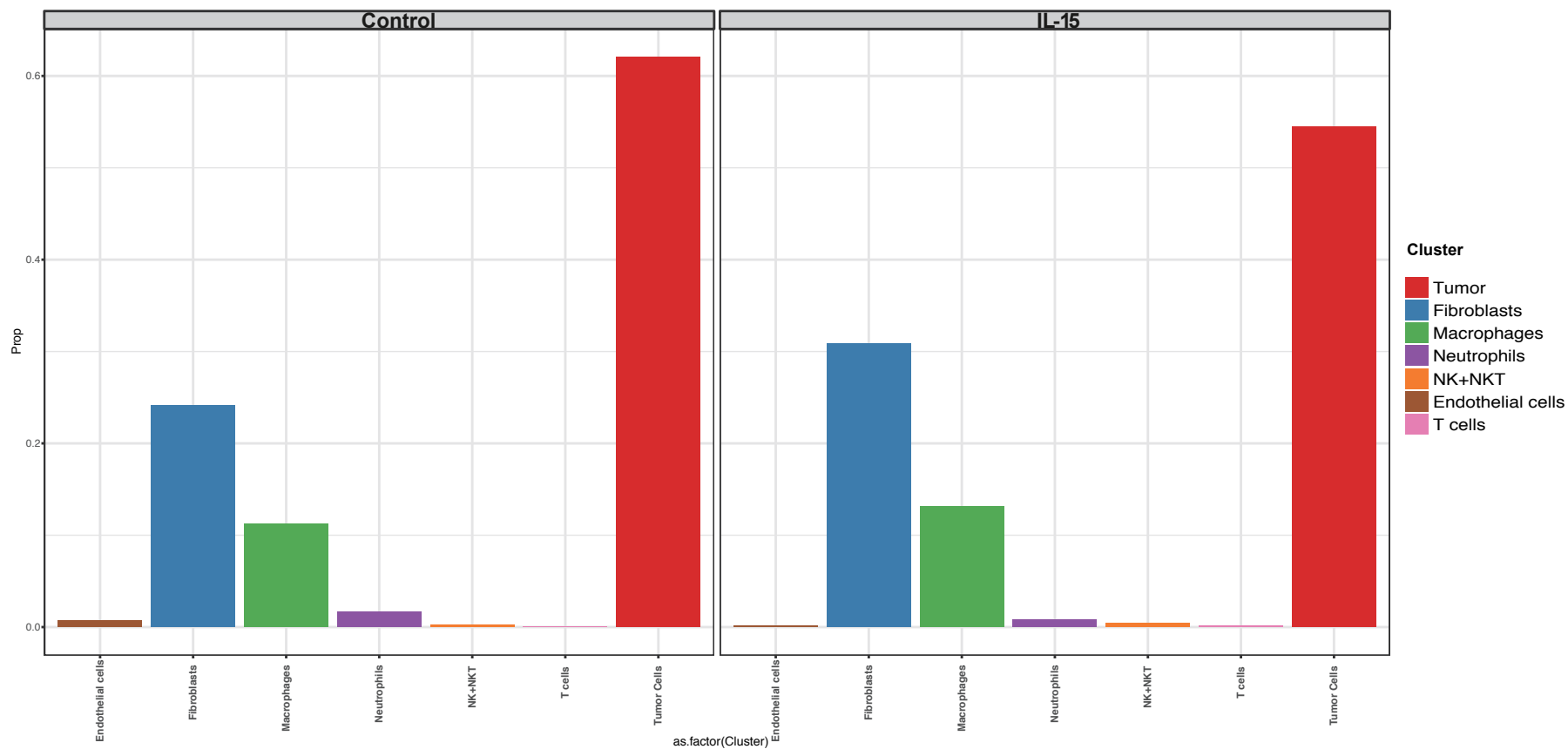

Supplement: Supplementary file 1 — Figure S1: Single‐cell analysis provided additional validation to the findings from flow cytometry. (A) The image of physical tumors collected from experiments. (B) Single cell analysis of the two major classes of immune cell types. (C) The cellular characteristics of the TME by single‐cell analysis. [file CRJ-19-e70117-s003.pdf]

# GO Annotation

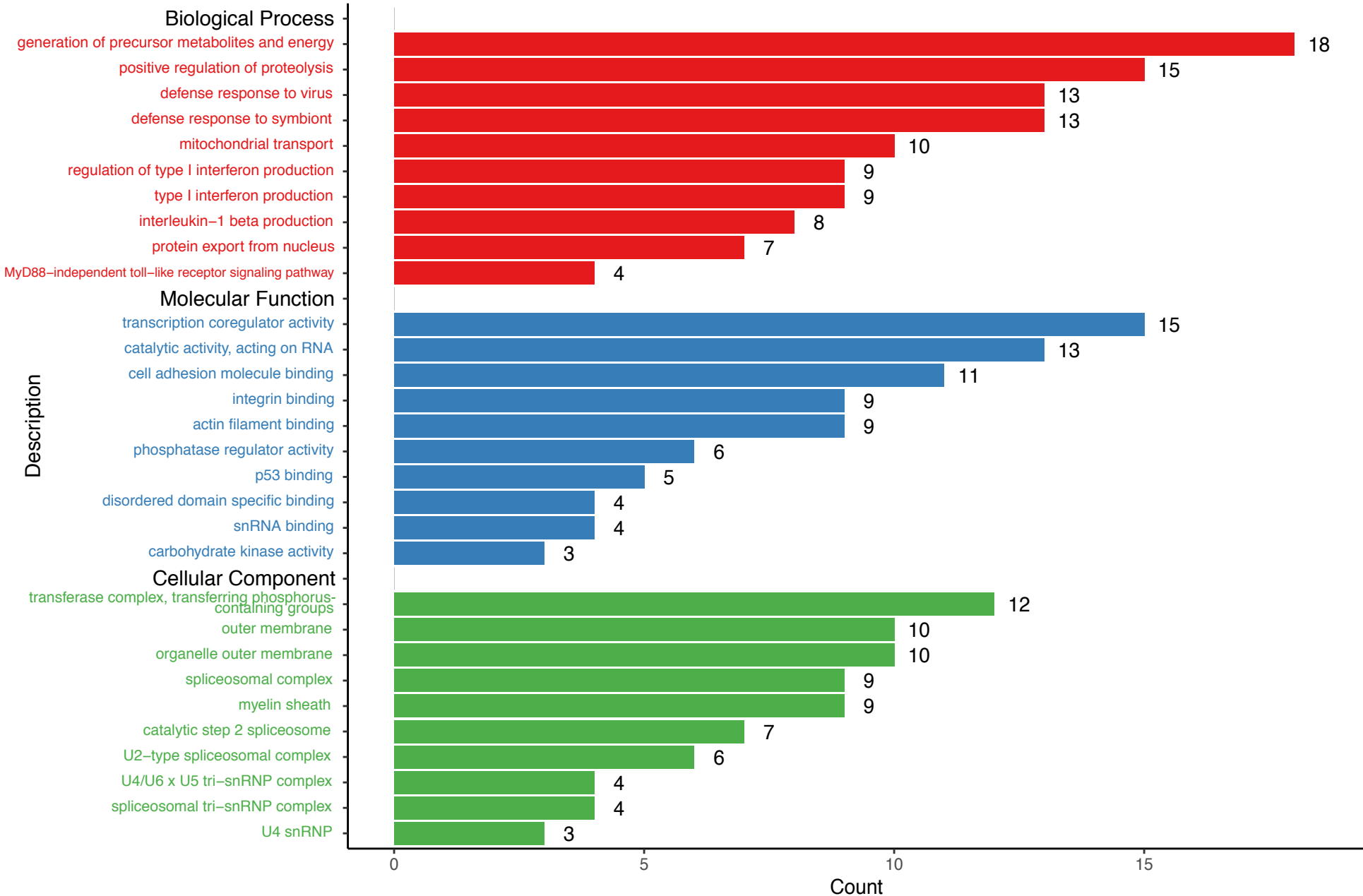

Supplement: Supplementary file 2 — Figure S2: GO analysis for endothelial cells. [file CRJ-19-e70117-s001.pdf]

# GO Annotation

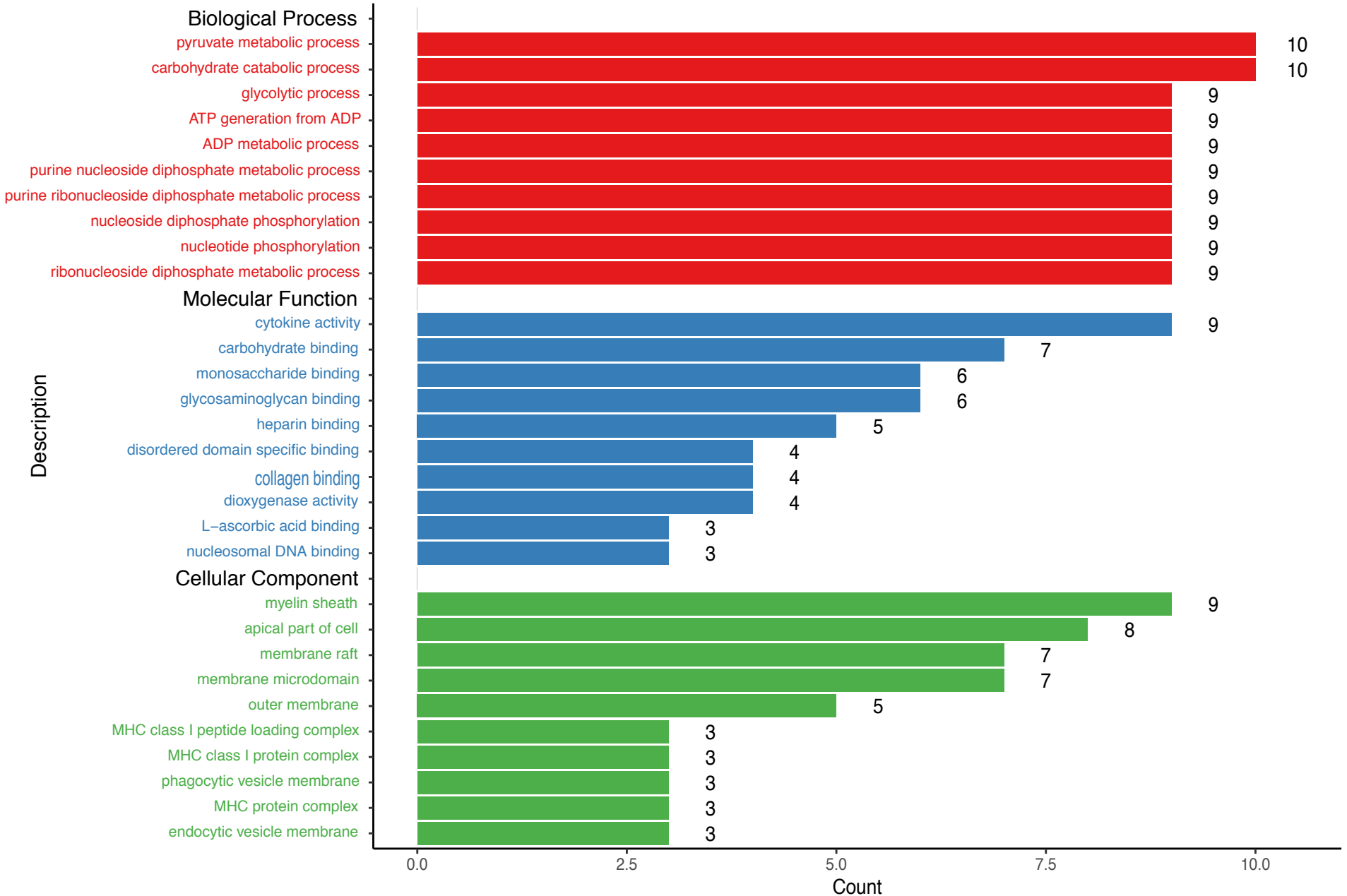

Supplement: Supplementary file 3 — Figure S3: GO analysis for fibroblasts. [file CRJ-19-e70117-s004.pdf]

# GO Annotation

Description

## Biological Process

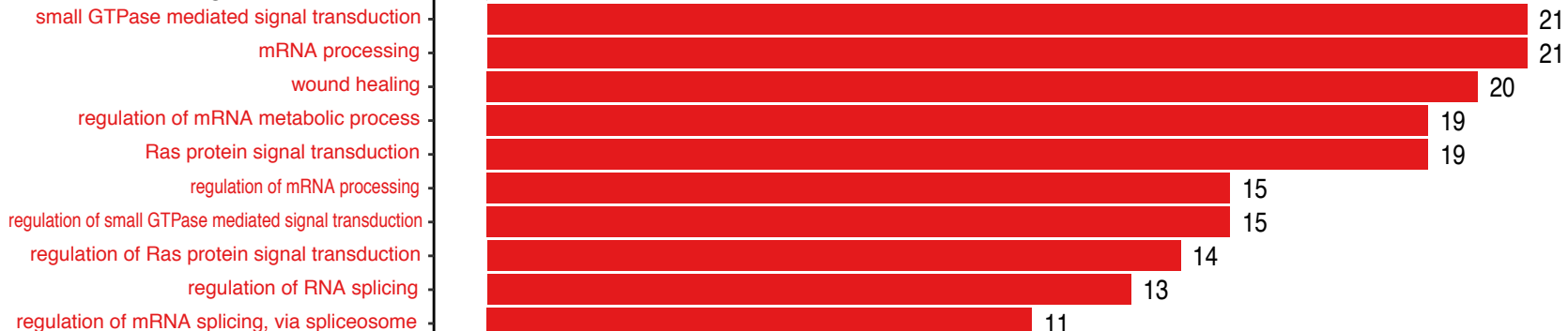

## Molecular Function

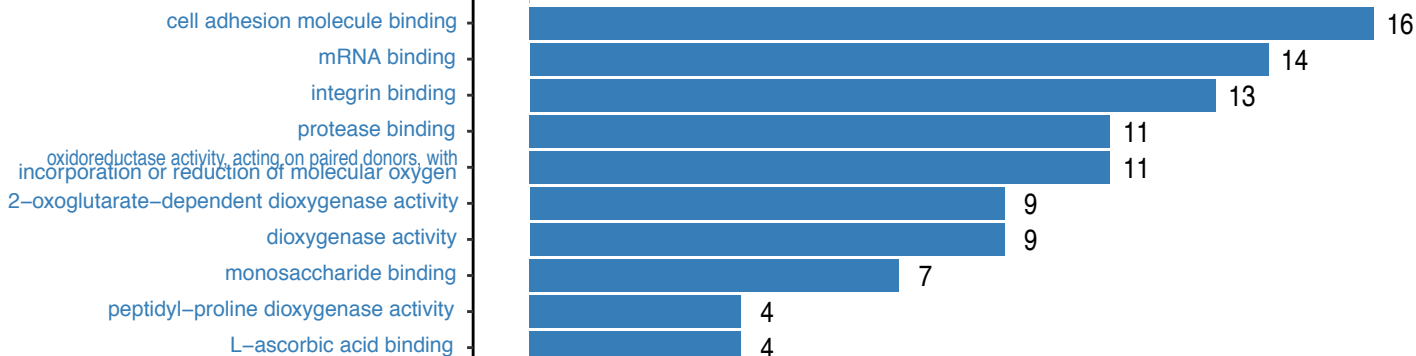

## Cellular Component

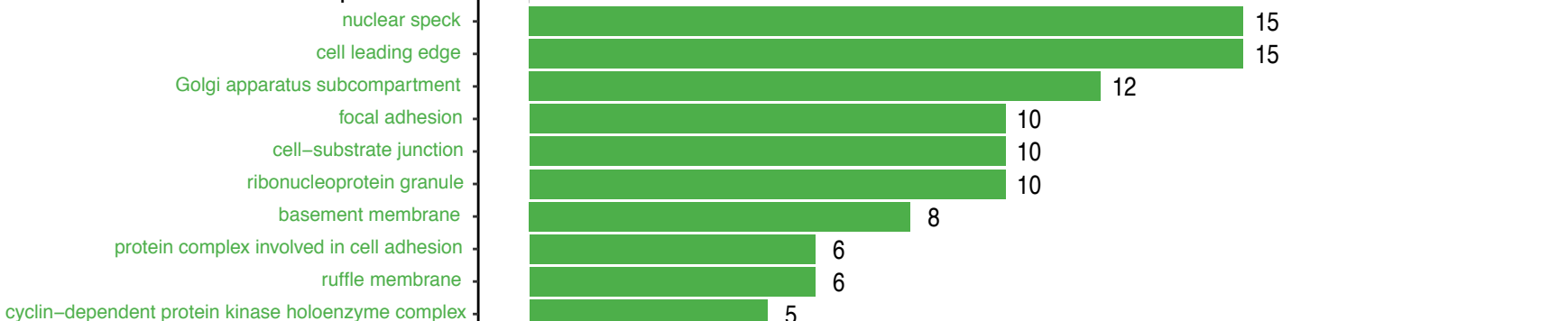

Count

Supplement: Supplementary file 4 — Figure S4: GO analysis for neutrophils. [file CRJ-19-e70117-s005.pdf]
