# Supplementary material for: IL‐15 Superagonist SHR‐1501 Enhances Immune Responses in Lung Cancer by Modulating Tumor Microenvironment
Source: Clin Respir J. 2025 Aug 13;19(8):e70117. doi: 10.1111/crj.70117 (PMC12344376; doi:10.1111/crj.70117)
Supplement: Supplementary file 5 — Data S1: Supplementary Information. [file CRJ-19-e70117-s002.docx]

**Supplementary figures:**

**Figure S1:** **Single-cell analysis provided additional validation to the findings from flow cytometry****.**

**A,** The image of physical tumors collected from experiments. **B,** Single cell analysis of the two major classes of immune cell types. **C,** The cellular characteristics of the TME by single-cell analysis.

**Figure S2: GO analysis for endothelial cells.**

**Figure S3: GO analysis for fibroblasts.**

**Figure S4: GO analysis for neutrophils.**
